# Tips and Tricks in Extracapsular Hip Fracture Fixation

**DOI:** 10.2174/1874325001711011218

**Published:** 2017-10-31

**Authors:** Ankit Desai, Josh Jacob, Alex Trompeter

**Affiliations:** 1 Specialist Registrar, Ashford and St. Peter’s Hospital, Surrey, UK; 2 Consultant Trauma Surgeon, St. Georges University Hospital, London, UK

**Keywords:** Extracapsular, Fracture neck of femur, Management, Dynamic hip screw, Extracapsular, Hip fracture

## Abstract

Aside from the decision-making in how to fix an extracapsular hip fracture, surgeons must be conversant with systems, implants and how to maximize their potential in the shortest operative time. We present a series of steps used in our unit when performing both DHS and intramedullary nail surgery to avoid the commonest problems and maximize our patients’ recovery potential.

## 1 INTRODUCTION

Surgical management of extracapsular neck of femur fractures is constantly evolving but, arguably, its perception as an operation for junior surgeons in training is not. This belies the potential complexity of the surgery and a very high level of morbidity and mortality. The surgical decision-making is sometimes difficult and poor fixation can lead to catastrophic outcomes. In this article we present some simple hints and tricks that can be employed in the management of these fractures, offering help to junior and senior surgeons alike.

### 1.1 Setting Up and Achieving Reduction

For both DHS and nailing, the patient is positioned supine on a fracture table with the contralateral leg positioned to permit access for the image intensifier. It can be useful to apply a modest degree of flexion to the injured leg to aid in reduction of the fracture. Whilst conventionally internal rotation is used to achieve reduction, too much can lead to exaggeration of any posterior-medial defect.

A residual apex-anterior deformity or lateralised femoral shaft may persist after initial reduction manoeuvres. An instrument may be passed over the anterior femoral neck and translated floorwards to reduce the neck angulation, conventionally through the incision to be used for the femoral neck screw Fig. (**1**). Alternatively, a ball spike may be employed through a percutaneous anterolateral incision [1]. Pressure exerted on the lateral femoral cortex can help reduce a lateralised femoral shaft Fig. (**2**). These reduction techniques have to be maintained throughout the procedure until the definitive implant is secure across the fracture.

## 2 ASSESSMENT OF REDUCTION

The routine method of assessing reduction is by obtaining the standard AP and lateral views. To aid decision-making in the reduction, we advocate obtaining a 15-20**°** oblique view, a true lateral which takes into account the anteversion of the femoral neck Fig. (**3**). This has been shown to significantly decrease the divergence between the axes of the lag screw and that of the femoral neck, and permits the use of the perineal post as an aiming point to position the screw centrally and mid-axially in head and neck of the femur respectively by referencing the surgeon’s hand to the floor, perpendicular to the post Fig. (**4**) [2]. Alternatively, a guide wire may be passed along the anterior cortex of the femoral neck to act as a visual cue for version.

## 3 TIPS AND TRICKS FOR THE DHS

The incision should be placed slightly posterior to the mid-axial line in the lateral plane, since the native femur is flatter postero-laterally and hence is an ideal site for the plate. The fascia of vastus lateralis may be incised slightly posteriorly, permitting the majority of the muscle to be reflected anteriorly for an exposure which does not sacrifice the intermuscular perforators.

**Fig. (5):**
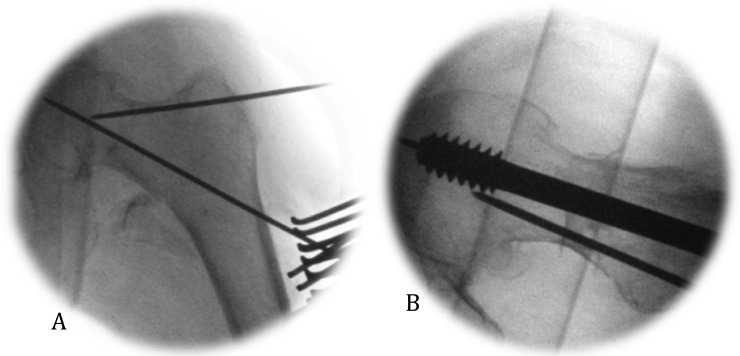
(A) Anti-rotation wire used at forty five degrees to the guide wire to facilitate stability during reaming and tapping of femoral neck.(B) Lateral radiograph of anti-rotation wire preventing rotation and fracture displacement during insertion of screw.

In the context of a basi-cervical fracture, a de-rotatation wire can be useful provided it is as far from the guide wire as possible in the femoral neck, to avoid fouling during reaming and screw implantation. A non-parallel trajectory is important to maximize resistance to torque (Fig. **5**).

While a locking plate offers 2.6 times more load to failure than its non-locked equivalent, it should be borne in mind that the classical mode of failure of the construct is cut-out of the screw from the head and hence achieving a Tip-Apex Distance (TAD) of less than 25mm is the most crucial objective [3-5].

## 4 TIPS AND TRICKS FOR INTRAMEDULLARY NAILING

If a satisfactory closed reduction is not achieved, an open reduction is required, which may be performed through minimal extensions of the operative incisions, aided by radiological identification of landmarks. A greater trochanteric entry point can be found at the intersection of a perpendicular line dropped from the anterior superior iliac spine and the extrapolation of the axis of the femur, remembering to take its anatomical bow into account. The deeper incision should split gluteus fibres in line, and be made in the imaginary line between skin incision and piriform fossa, rather than immediately deep to skin. This may be achieved by directing the scalpel towards the greater trochanter with an oblique direction of advancement. Common pitfalls to be avoided are placing the skin incision too distal or the gluteus split too proximal, both of which lead to sub-optimal access to the intramedullary canal.

The entry point for the guide wire into the greater trochanter should be made under radiological guidance, aiming slightly medial to the tip of the greater trochanter. An excessively lateral entry point can cause lateralisation of the femoral shaft and varus mal-reduction.

The jig may be used as a reduction tool once the nail is past the fracture and can help reduce a varus direction of the guide wire for the compression screw. Pressure on the distal end of the jig, producing a valgus force, is the best way of achieving this.

Intra-operative peri-prosthetic fracture is generally avoidable by meticulous screening, awareness of the radii of curvature of the implant and femur and attention to reaming before passing the nail. Should it occur, in a true proximal femoral fracture a short nail is a viable fallback option, with minimally invasive bridge plate fixation of the peri-prosthetic fracture and appropriately protective weight-bearing status.

Although aiming devices are now available for some modern nailing systems, fluency in the use of the “perfect circle” technique offers rapid and reproducible results. Once the distal locking holes are seen on the image intensifier as perfect circles, implying the beam of the image intensifier is directly in line with them, a stab incision can be made and then the drill lined up with the beam to drill through the cortex and past the nail Fig. (**6**). Depending on experience and operator preference, the drill bit may then be de-coupled and left in situ prior to obtaining a view demonstrating it to be through the nail (occluding the hole) and subsequently drilling the far cortex.

## CONCLUSION

One of the key steps in managing these fractures is deciding which implant to use but, after this, competence and awareness of pitfalls are key to deliver the lowest morbidity results and the highest potential recovery.

## Figures and Tables

**Fig. (1):**
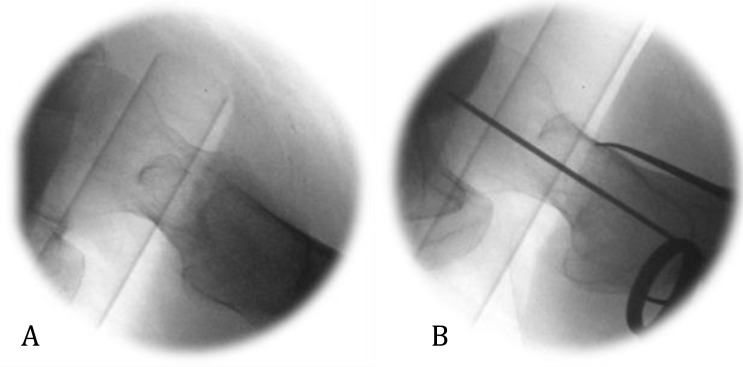
Lateral radiograph of right hip showing (A) apex anterior angulation and (B) correction with downward pressure through a lever placed over the anterior femoral neck.

**Fig. (2):**
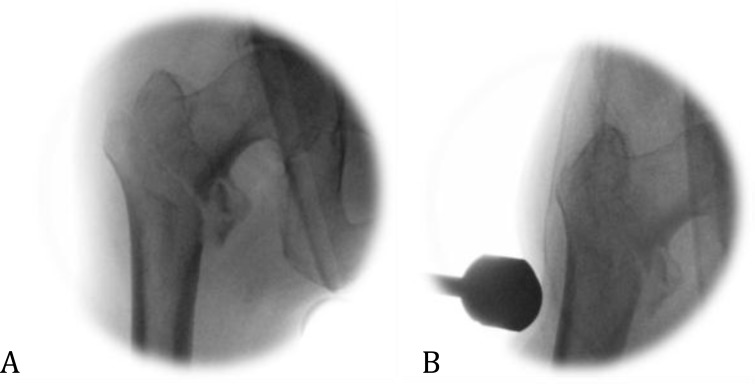
AP radiographs of a right hip showing (A) a laterally displaced fracture (B) the fracture reduced with lateral pressure applied firmly over the fracture site.

**Fig. (3):**
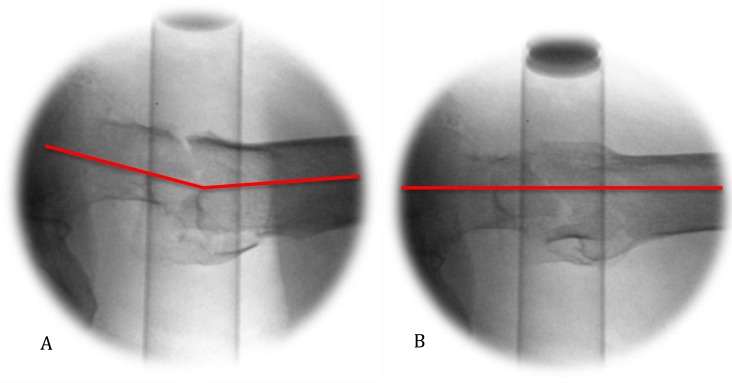
Lateral radiographs of the right femoral neck taken at (A) neutral and (B) 20 degree angulation of beam.

**Fig. (4):**
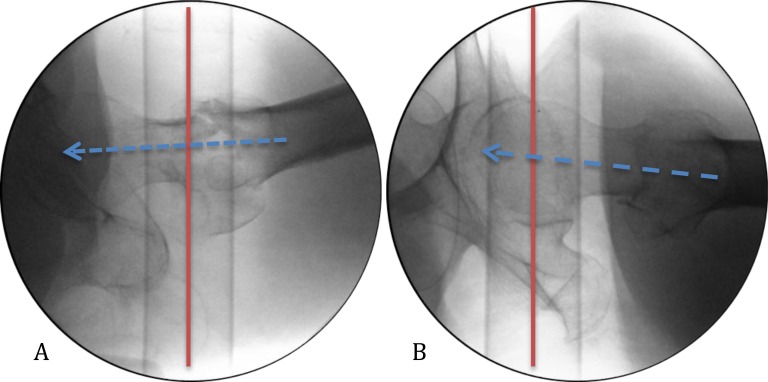
Proximal femur lateral view with peroneal posts visible. Trajectory of guide wire (blue arrow) can be assessed compared to peroneal post which is 90° to the floor (red line). (A) shows a downwards trajectory. (B) shows an upwards trajectory. This technique allows the surgeon to understand the lateral trajectory while inserting the guide wire which is routinely performed with AP fluroscopy.

**Fig. (6):**
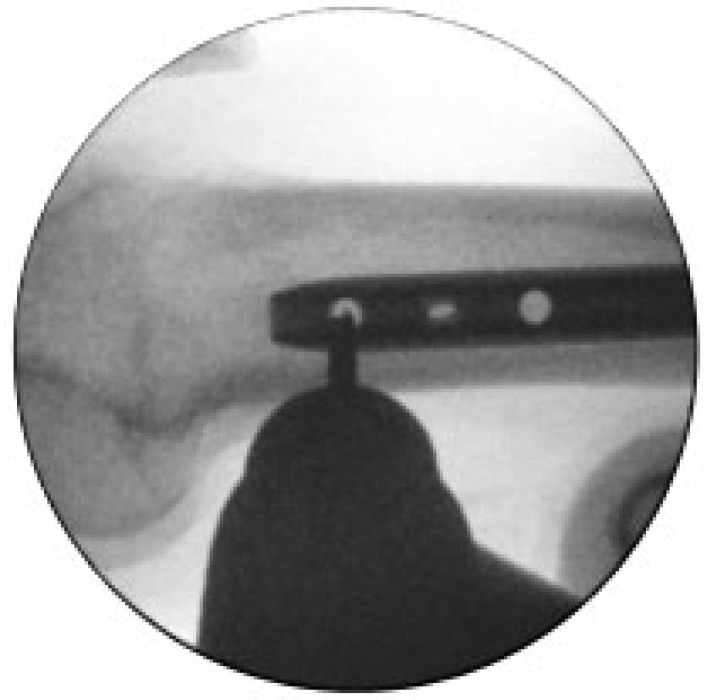
Distal femoral locking holes shown with perfect circles. Drill piece located directly over distal hole. Oblique locking screw hole (middle hole) shows oval rather than circle indicating that the hole trajectory is not parallel to C-arm. Horizontal orientated oval helps determine correction required in C-arm to obtain perfect circle.
